# Targeting PI3K/Akt/mTOR Signaling in Cancer

**DOI:** 10.3389/fonc.2014.00064

**Published:** 2014-04-14

**Authors:** Camillo Porta, Chiara Paglino, Alessandra Mosca

**Affiliations:** ^1^Medical Oncology, Fondazione I.R.C.C.S. Policlinico San Matteo University Hospital Foundation, Pavia, Italy; ^2^Medical Oncology, Maggiore della Carità Hospital, University of Eastern Piedmont “A. Avogadro”, Novara, Italy

**Keywords:** PI3K, Akt, mTOR, inhibitors, temsirolimus, everolimus, ridaforolimus, novel agents

## Abstract

The phosphatidylinositol-3-kinase (PI3K)/Akt and the mammalian target of rapamycin (mTOR) signaling pathways are two pathways crucial to many aspects of cell growth and survival, in physiological as well as in pathological conditions (e.g., cancer). Indeed, they are so interconnected that, in a certain sense, they could be regarded as a single, unique pathway. In this paper, after a general overview of the biological significance and the main components of these pathways, we address the present status of the development of specific PI3K, Akt, and mTOR inhibitors, from already registered medicines to novel compounds that are just leaving the laboratory bench.

## Introduction

**Figure 1:**
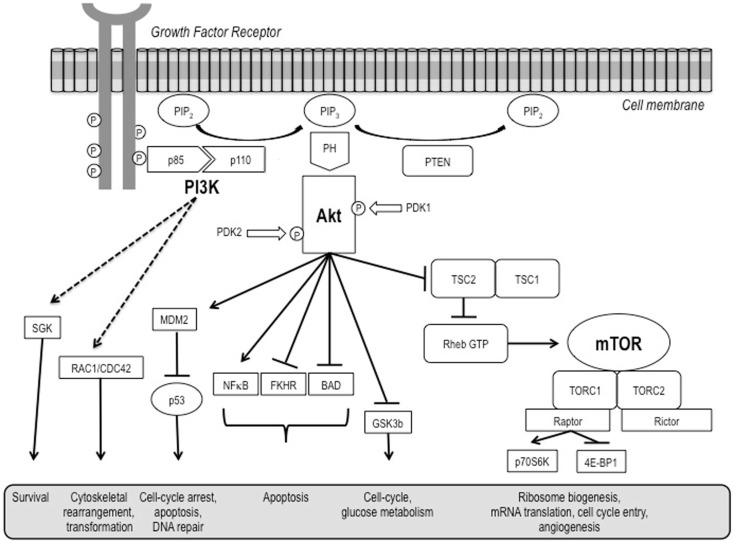
**A schematic representation of the PI3K/Akt/mTOR pathway**.

The phosphatidylinositol-3-kinase (PI3K)/Akt and the mammalian target of rapamycin (mTOR) signaling pathways are both crucial to many aspects of cell growth and survival, in physiological as well as in pathological conditions. They are so interconnected that, in a certain sense, they could be regarded as a single, unique pathway (Figure 1) that, in turn, heavily interacts also with many other pathways, including that of the hypoxia inducible factors (HIFs).

The PI3K/Akt pathway is a key regulator of survival during cellular stress (1). Since tumors exist in an intrinsically stressful environment (characterized by limited nutrient and oxygen supply, as well as by low pH), the role of this pathway in cancer appears to be crucial.

Mammalian target of rapamycin is a serine/threonine kinase ubiquitously expressed in mammalian cells (2). It picks up and integrates signals initiated by nutrient intake, growth factors, and other cellular stimuli to regulate downstream signaling and protein synthesis. Through its downstream effectors, 4EBP1 and P70S6 kinase (S6K), it is involved in the initiation of ribosomal translation of mRNA into proteins necessary for cell growth, cell cycle progression, and cell metabolism.

Somatic mutations and/or gains and losses of key genes are among a number of genetic alterations affecting these pathways in a number of different solid and hematological tumors [including big killers such as breast and colon cancer, as well as relatively less frequent neoplasms such as neuroendocrine tumors (NETs), kidney cancer, and some lymphomas]. The activation of the PI3K/Akt/mTOR pathway results in a profound disturbance of control of cell growth and survival, which ultimately leads to a competitive growth advantage, metastatic competence, angiogenesis, and therapy resistance.

Thus, this complex pathway has been taken into consideration as one of the most attractive targets for the development of anticancer agents (3, 4).

## PI3K Structure and Functions

Phosphatidyl-inositol-3-kinases (PI3Ks) constitute a lipid kinase family characterized by the capability to phosphorylate inositol ring 3′-OH group in inositol phospholipids (5). Class I PI3Ks are heterodimers composed of a catalytic (CAT) subunit (i.e., p110) and an adaptor/regulatory subunit (i.e., p85).

This class is further divided into two subclasses: subclass IA (PI3Kα, β, and δ), which is activated by receptors with protein tyrosine kinase activity, and subclass IB (PI3Kγ), which is activated by receptors coupled with G proteins (5).

Activation of growth factor receptor protein tyrosine kinases results in autophosphorylation on tyrosine residues. PI3K is then recruited to the membrane by directly binding to phosphotyrosine consensus residues of growth factor receptors or adaptors through one of the two SH2 domains in the adaptor subunit. This leads to allosteric activation of the CAT subunit. In a few seconds, PI3K activation leads to the production of the second messenger phosphatidylinositol-3,4,5-triphosphate (PI3,4,5-P_3_) from the substrate phosphatidylinositol-4,4-bisphosphate (PI-4,5-P_2_). PI3,4,5-P_3_ then recruits a subset of signaling proteins with pleckstrin homology (PH) domains to the membrane, including protein serine/threonine kinase-3′-phosphoinositide-dependent kinase 1 (PDK1) and Akt/protein kinase B (PKB) (5, 6). Akt/PKB, on its own, regulates several cell processes involved in cell survival and cell cycle progression.

As far as cell survival is concerned, Akt/PKB can inactivate pro-apoptotic factors such as Bad and Procaspase-9, as well as the Forkhead family of transcription factors that induce the expression of other pro-apoptotic factors, such as Fas-ligand (FasL) (7, 8). Akt/PKB activation has been related to an increased resistance of prostate cancer cells to apoptosis mediated by tumor necrosis factor (TNF)-related apoptosis inducing ligand (TRAIL)/APO-2L (9). Finally, Akt/PKB also activates the IκB kinase (IKK), a positive regulator of the survival factor NFκB. Notably, a strong biological link between the NFκB and the PI3K/Akt pathways in the modulation of anti-apoptotic effects in lymphoma cells exposed to the irreversible inhibitor of the activation of NFκB and the phosphorylation of IκBα BAY11-7085 has been also shown (10).

As for cell cycle progression and cell growth, several targets of Akt are involved in protein synthesis, glycogen metabolism, and cell cycle regulation (6), including the same mTOR, glycogen synthase kinase-3 (GSK3), insulin receptor substrate-1 (IRS-1), the cyclin-dependent kinase inhibitors p21^CIP1/WAF1^ and p27^KIP1^, and possibly also Raf-1, a member of the MAPK pathway. With regard to GSK3, Akt/PKB triggers a network that positively regulates G1/S cell cycle progression through inactivation of GSK3-β, leading to increased cyclin D1, and inhibition of the Forkhead family transcription factors and the tumor suppressor tuberin (TSC2), ultimately resulting in the reduction in p27Kip1 (11).

## Akt Structure and Functions

Akt kinases belong to the AGC kinase family, related to AMP/GMP kinases and protein kinase C. They consist of three conserved domains, an N-terminal PH domain, a central kinase CAT domain, and a C-terminal extension (EXT) containing a regulatory hydrophobic motif (HM) (12). Among the Akt isoforms, the PH domains are ~80% identical and ~30% identical to PH domains in pleckstrin and other proteins. The linker (LINK) region connecting the PH domain to the CAT domain is poorly conserved among the Akt isoforms (17–46% identical) and has no significant homology to any other human protein (12). The consensus CAT domain is ~90% identical among the Akt isoforms and is closely related the PKC, PKA, SGK, and S6 subfamilies of the AGC kinase family (12). The C-terminal EXT is ~70% identical among the Akt isoforms and is most closely related to the PKC family (12).

## mTOR Structure and Functions

Mammalian target of rapamycin is a key protein evolutionarily conserved from yeast to man and is essential for life. Indeed, embryonic mutations in mTOR proved to be lethal.

In normal cells, mTOR activity is controlled by positive and negative upstream regulators (13). Positive regulators include growth factors and their receptors, such as insulin-like growth factor-1 (IGF-1) and its cognate receptor IFGR-1, members of the human epidermal growth factor receptor (HER) family and associated ligands, and vascular endothelial growth factor receptors (VEGFRs) and their ligands, which transmit signals to mTOR through the PI3K-Akt. Negative regulators of mTOR activity include phosphatase and tensin homolog (PTEN) that inhibits signaling through the PI3K-Akt pathway, and tuberous sclerosis complex (TSC) 1 (hamartin) and TSC2 (tuberin). Phosphorylation of TSC2 by Akt releases its inhibitory effect on mTOR and up-regulates mTOR activity. Another negative regulator, LKB1, is in an energy-sensing pathway upstream of TSC (14).

Mammalian target of rapamycin activity is carried out by two distinct complexes: mTORC1 and mTORC2.

The mTORC1 complex is made up of mTOR, Raptor, mLST8, and PRAS40. It is extremely sensitive to rapamycin and thus represents the target of first-generation mTOR inhibitors. It also activates S6K and inactivates 4EBP1, leading to protein translation and cell growth (13).

The mTORC2 complex is composed of mTOR, Rictor, Sin1, and mLST8. It is less sensitive to rapamycin and its role in normal cell function and oncogenesis has not been well clarified. However, it is known to activate Akt, thereby promoting cell proliferation and survival. The canonical pathway of mTOR activation depends on mitogen-driven signaling through PI3K/Akt, although alternative non-Akt dependent activation through the Ras/MEK/ERK pathway is now recognized (15).

Altogether, mTOR activation leads to increased synthesis of multiple proteins. These include several that have been implicated in the pathogenesis of multiple tumors, e.g., cyclin D1, which allows progression of cells through the cell cycle (16), and HIF, which drive the expression of pro-angiogenic growth factors such as VEGF (17).

## PTEN as a Regulator of the PI3K/Akt/mTOR Pathway

The PTEN deleted on chromosome 10 (PTEN) is a key molecule downstream of the PI3K/Akt pathway. This phosphatase, endowed with dual activity on both lipids and proteins, acts as a tumor suppressor by inhibiting cell growth and enhancing cellular sensitivity to apoptosis and anoikis, i.e., an epithelial cell-peculiar type of apoptosis triggered by alterations in integrin–extracellular matrix interactions (18).

Phosphatase and tensin homolog is frequently mutated in several advanced human cancers. In addition, PTEN mutations in germ cell lines result in the rare hereditary syndrome known as Cowden’s disease, which is associated with a higher risk of different cancers, including breast, thyroid, and endometrial cancers (19).

The main lipid substrate of PTEN is PI3,4,5-P_3_, and indeed PTEN acts as a negative regulator of PI3K/Akt signaling. Thus, loss of PTEN activity leads to permanent PI3K/Akt pathway activation.

## Development of mTOR Inhibitors as Anticancer Agents

Rapamycin (sirolimus), an antifungal agent with immunosuppressive properties, was first isolated in 1975 from the soil of the island of Rapa Nui or Easter Island (20). Already back in the 1980s, when tested against a panel of human cancer cell lines, rapamycin showed a broad anticancer activity (21). However, clinical development of rapamycin as an anticancer agent was hampered by unfavorable pharmacokinetic properties (22).

The relatively recent development of rapamycin analogs endowed with a more favorable pharmacokinetic profile, i.e., temsirolimus, everolimus, and ridaforolimus (a.k.a. deforolimus), opened up the present era of mTOR inhibitors as anticancer agents.

**Figure 2:**
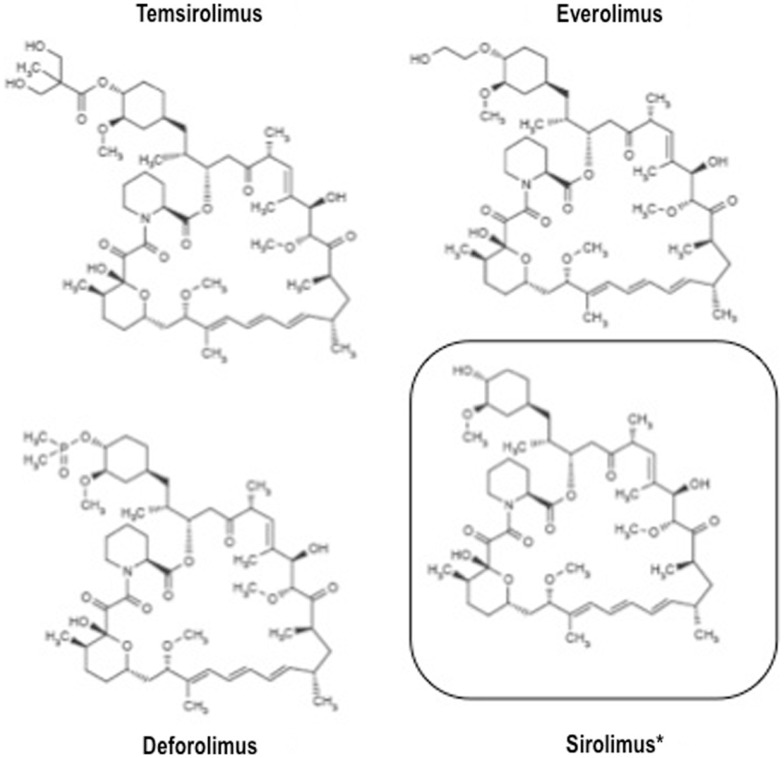
**Structure of clinically available mTOR inhibitors**.

All these agents have similar structure (Figure 2) and mechanism of action, but different pharmacokinetic properties. Indeed, all these drugs are small molecule inhibitors that function intracellularly, forming a complex with the FK506 binding protein-12 (FKBP-12) that is then recognized by mTOR. The resulting complex prevents mTOR activity, leading to inhibition of cell cycle progression, survival, and angiogenesis. Notably, all these inhibitors are similar to the parental compound rapamycin in that they affect only mTORC1, and not mTORC2 (22).

## Temsirolimus: Phase III Trials

Temsirolimus is a pro-drug whose primary active metabolite is rapamycin. Temsirolimus is administered intravenously on a once-weekly schedule (23). It has been approved for the treatment of patients with advanced renal cell carcinoma (RCC) with poor prognostic features, and of mantle cell lymphoma (MCL) patients.

### Renal cell carcinoma

Temsirolimus registration in RCC was obtained on the basis of the positive results of a randomized, controlled, phase III trial of temsirolimus, interferon-α, or a combination of the two (24). In this study, 626 patients with previously untreated, poor-prognosis, metastatic RCC were randomized to receive 25 mg of intravenous (i.v.) temsirolimus weekly, 3 MU of interferon-α (with an increase to 18 MU) subcutaneous (s.c.) three times weekly, or a combination-therapy with 15 mg of temsirolimus weekly plus 6 MU of interferon-α three times weekly. Overall survival (OS) was the primary endpoint of the trial. Patients who received temsirolimus alone had longer OS and progression-free survival (PFS) than patients who received interferon-α alone, while there was no significant difference in OS between the combination-therapy group and the interferon group. Indeed, median OS in the temsirolimus group, the interferon-α group, and the combination-therapy group were 10.9, 7.3, and 8.4 months, respectively (24).

### Mantle cell lymphoma

As far as MCL is concerned, the pivotal registration trial was a phase III trial evaluating two dose regimens of temsirolimus in comparison with single-agent therapy in relapsed or refractory disease (investigator’s choice) (25). Sixty-two patients with relapsed or refractory MCL were randomly assigned to receive one of two temsirolimus regimens: 175 mg weekly for 3 weeks followed by either 75 mg (175/75 mg) or 25 mg (175/25 mg) weekly, or investigator’s choice therapy from approved options. PFS was the primary endpoint of the study. Median PFS was 4.8, 3.4, and 1.9 months for the temsirolimus 175/75, 175/25 mg, and investigator’s choice groups, respectively (25). Patients treated with temsirolimus 175/75 mg had significantly longer PFS than those treated with investigator’s choice therapy [*p* = 0.0009; hazard ratio (HR) = 0.44]. Furthermore, objective response rate (ORR) was significantly higher in the 175/75-mg group (22%) compared with the investigator’s choice group (2%; *p* = 0.0019), while there was no statistical difference in OS (25).

## Ridaforolimus: Phase III Trial

Ridaforolimus is not a pro-drug, but like temsirolimus, it was originally administered intravenously on an intermittent schedule, while an oral formulation has also been subsequently developed (26, 27).

### Maintenance treatment for adult soft tissue and bone sarcomas

Recently, a large randomized, placebo-controlled, phase III trial was carried out aiming to evaluate ridaforolimus activity as a maintenance treatment in advanced sarcomas (28). In this study, 711 patients with metastatic soft tissue or bone sarcomas who achieved an objective response or at least a stable disease after standard chemotherapy were randomly assigned to receive ridaforolimus 40 mg or placebo once per day, per oral administration (o.s.) for 5 days every week. The primary endpoint was PFS. Overall, ridaforolimus treatment led to a modest, although statistically significant, improvement in PFS compared with placebo (17.7 vs. 14.6 weeks; HR: 0.72; 95% CI: 0.61–0.85; *p* = 0.001) (28).

## Everolimus: Phase III Trials

Everolimus is another orally available mTOR inhibitor that is usually administered on a continuous daily schedule (even though a weekly schedule has been also tested, especially for combination regimens) (29).

### Renal cell carcinoma

Everolimus has recently been approved by the US Food and Drug Administration (FDA) and European Medicines Agency (EMA) for the treatment of advanced RCC after failure of treatment with Sunitinib and/or Sorafenib, following the presentation of the results of the RECORD-1 trial.

RECORD-1 was a phase III double-blind, randomized, placebo-controlled trial aimed at evaluating the activity of everolimus in patients whose disease had progressed under treatment with one or two VEGFR tyrosine kinase inhibitors (TKIs) (30). A total of 416 patients were enrolled and stratified according to the number of previous treatments [Sorafenib or Sunitinib (1 TKI) vs. Sorafenib as well as Sunitinib (2 TKIs)] and prognostic risk group. Patients were then randomized in the ratio of 2 to 1 to receive everolimus (given at the standard dose of 10 mg daily, per o.s.) plus best supportive care (BSC), or to placebo plus BSC. After the second interim analysis, the study was terminated since the pre-specified efficacy endpoint had been met (30). Indeed, at the final trial analysis, everolimus proved able to significantly improve PFS when compared to placebo: 4.9 vs. 1.9 months, respectively (HR: 0.33; 95% CI: 0.25–0.43; *p* < 0.001) (31). Furthermore, everolimus significantly increased median PFS in each risk group regardless of whether patients had received one or two prior TKIs (32), had stopped prior therapy for intolerance (33), or of patient age (34).

### Neuroendocrine tumors

As most NETs are hypervascular (35) and synthesize and secrete high levels of VEGF-A (36, 37), targeted (such as everolimus and sunitinib) and untargeted (such as somatostatin analogs, interferon-α, and thalidomide) therapies, with certain or possible anti-angiogenic properties, have been tested in metastatic NET.

Everolimus, in association with octreotide LAR, first demonstrated a promising antitumor activity in a phase II trial with 30 low- to intermediate-grade NET (carcinoids) patients, showing 17% of partial remission and 80% of stable disease, added to a median PFS of 15.7 months (38).

The open-label, phase II trial RADIANT-1 enrolled 160 advanced, low- or intermediate-grade pancreatic NET (pNET) patients, with progressive (according to RECIST criteria) disease during or after cytotoxic chemotherapy (39). One hundred and fifteen patients were assigned to everolimus 10 mg/day o.s., and 45 patients were submitted to everolimus 10 mg/day o.s. + octreotide LAR 30 mg/28 days intramuscular (i.m). The response rates were 9.6% in the everolimus arm and 4.4% in the everolimus + octreotide LAR group. Median PFS by central radiology review were 9.7 months for patients receiving everolimus and 16.7 months for those receiving the combination (39). Furthermore, high baseline levels of chromogranin A and neuron-specific enolase circulating neuroendocrine markers were associated with shorter median PFS and OS (40).

The favorable results of these previous phase II trials were then confirmed in two international, multicenter, randomized, placebo-controlled, phase III studies (RADIANT-2 and RADIANT-3).

In the RADIANT-2 study (41), 429 patients with advanced progressive midgut NETs were randomized to receive everolimus 10 mg/day plus octreotide LAR 30 mg/month or octreotide LAR plus placebo. A clinically significant improvement in PFS was recorded in the everolimus arm compared with octreotide LAR/placebo arm (16.4 vs. 11.3 months, respectively), even though the pre-defined threshold for statistical significance was not reached, according to central radiological reading (41). A subsequent multivariate analysis and the local radiological reading sustained the efficacy of everolimus. Furthermore, a subgroup analysis underlined some potential primary tumor sites in particular that could benefit, such as bronchial/lung NETs or colonic NETs (42). Nevertheless, the precise therapeutic activity of everolimus in advanced progressive midgut NETs remained to be defined (43).

In RADIANT-3 (44), the largest clinical trial conducted in pNET patients, 410 patients with advanced pNET and progressive disease were randomly assigned to treatment with oral everolimus 10 mg/day or placebo. Octreotide LAR was administered at the discretion of the investigator. Everolimus was associated with an improvement in median PFS compared with placebo (11.0 vs. 4.6 months, respectively; *p* < 0.0001), and with an overall tumor response rate of 5% (44). The most common drug-related toxicities were G1–2 stomatitis or aphthous ulceration (44). Furthermore, everolimus therapy correlated with a reduction in VEGF pathway markers, such as soluble VEGF receptor 2 and placental growth factor, suggesting an anti-angiogenic activity of everolimus in pNET patients (45).

Even though everolimus evidently inhibited tumor growth and delayed time-to-progression, the percentages of progression events (i.e., appearance of new metastasis as the only cause of progression, appearance of new metastasis concurrent with progression of preexisting metastases, lesion growth at baseline without new metastases appearing) in the two arms (everolimus, placebo) were similar, suggesting that everolimus delayed tumor progression without affecting the pattern of progression in advanced pNET patients (46).

Following the RADIANT-3, in 2011, everolimus was approved for the treatment of progressive pNETs, but its efficacy in other NETs remains uncertain. Given that RADIANT-2, including 51% of small intestinal carcinoids, failed to achieve its primary endpoint, a placebo-controlled trial with everolimus as monotherapy in progressive gastro-intestinal and lung carcinoids (RADIANT-4) is now ongoing. In the meantime, NCCN guidelines recommend everolimus among several therapeutic options in clinically significant progressive NETs, and by ENETS guidelines in non-pNETs with progressive disease after all other medical treatments (43).

### Breast cancer

Recently, the combination of everolimus with the aromatase inhibitor Exemestane has been evaluated in a randomized, phase III trial, since a large amount of evidence supported the hypothesis that aberrant signaling through the mTOR pathway is associated with resistance to endocrine therapies (47).

In the BOLERO-2 phase III trial (48), 724 patients with hormone-receptor-positive advanced breast cancer who recurred or progressed while receiving treatment with a non-steroidal aromatase inhibitor in the adjuvant or metastatic setting, were randomized two to one to receive everolimus and exemestane or exemestane and placebo. PFS was the primary endpoint of the study. A pre-planned interim analysis was performed by an independent data and safety monitoring committee after 359 PFS events were observed. At the time of this analysis, median PFS assessed by the Investigators PFS was 6.9 months with everolimus plus Exemestane and 2.8 months with Placebo plus Exemestane (HR: 0.43; 95% CI: 0.35–0.54; *p* < 0.001), while centrally assessed PFS was 10.6 and 4.1 months, respectively, again in favor of the everolimus-containing combination (HR: 0.36; 95% CI: 0.27–0.47; *p* < 0.001) (48).

### Other indications

Everolimus also has an established role in the treatment of two rare conditions: renal angiomyolipomas associated with TSC or lymphangioleiomyomatosis, as well as TSC-related subependymal giant cell astrocytoma (SEGA), both characterized by constitutive activation of the mTOR pathway (49).

In the EXIST-2 double-blind, placebo-controlled, phase III trial (50), 118 patients with at least one angiomyolipoma 3 cm or larger in its longest diameter and a definite diagnosis of TSC or sporadic lymphangioleiomyomatosis were randomly assigned 2:1 to either everolimus 10 mg/day or Placebo. The primary efficacy endpoint of the study was the proportion of patients with confirmed angiomyolipoma response of an at least 50% reduction in total volume of target angiomyolipomas relative to baseline. Response rate (as defined above) was 42% [33 of 79 (95% CI: 31–53%)] for everolimus and 0% [0 of 39 (95% CI: 0–9%)] for placebo (*p* < 0.0001) (50), thus suggesting the usefulness of everolimus in this setting.

Similarly, in the EXIST-1 double-blind, placebo-controlled, phase III trial (51), 117 patients were randomized in a 2:1 ratio to everolimus 4.5 mg/m^2^/day (titrated to achieve blood trough concentrations of 5–15 ng/mL) or Placebo. Eligible patients had a definite diagnosis of TSC and at least one lesion with a diameter of 1 cm or greater, and either serial growth of an SEGA, a new lesion of 1 cm or greater, or new or worsening hydrocephalus. The primary endpoint of this study was the proportion of patients with confirmed response, i.e., a reduction in target volume of 50% or greater relative to baseline in SEGA. Twenty-seven (35%) patients in the everolimus group had an at least 50% reduction in SEGA volume as compared to none in the Placebo group (95% CI: 15–52; *p* < 0.0001) (51).

Taken together, these two studies suggest the possibility that everolimus might represent a disease-modifying treatment also for other aspects of tuberous sclerosis.

## The Safety Profile of mTOR Inhibitors

Adverse events observed in patients treated with mTOR inhibitors are fairly constant, irrespective of each specific indication. They include cutaneous and mucosal events (i.e., stomatitis and skin rash), pulmonary dysfunction (non-infectious pneumonitis), metabolic abnormalities (elevated blood levels of glucose, cholesterol, and triglycerides), as well immune-related events (i.e., increased incidence of infections) (52). As far as the risk of infections is concerned, we should not forget that mTOR inhibitors were first developed as immune suppressive agents and are still widely used as such in the transplantation setting.

Metabolic and immune-related adverse events are clearly on-target effects of mTOR inhibition, while cutaneous and mucosal effects may have a less direct association with mTOR inhibition, although inhibition of mTOR-mediated growth and tissue repair and/or immune dysregulation have been proposed to be a factor in mucosal epithelia with high turnover (53, 54).

In general, the incidences of key class-effect adverse events in the three largest phase III trials of everolimus (i.e., RECORD-1 in RCC, RADIANT-3 in pNET, and BOLERO-2 in hormone receptor-positive, HER2-negative, advanced breast cancer) were comparable (30, 44, 48), as summarized in Table 1.

**Table 1:** **Incidence of the main adverse events (all grades and grade 3/4) reported in the three largest phase III studies of everolimus in advanced solid tumors (RCC, pNET, and breast cancer)**.

	RCC (27)		pNET (41)		Breast cancer (46)	
	Everolimus + BSC^a^ (*n* = 274)		Everolimus monotherapy (*n* = 204)		Everolimus + exemestane (*n* = 482)	
	All grades (%)	Grade 3/4 (%)	All grades (%)	Grade 3/4 (%)	All grades (%)	Grade 3/4 (%)
Stomatitis	44	4	64	7	59	8
Rash	29	1	49	<1	39	1
Non-infectious pneumonitis	14	4	17	2	16	3
Hyperglycemia	57	15	13	5	14	5
Infections	37	10	23	2	50	5

*^a^BSC, best supportive care*.

## The Development of PI3K and Akt Inhibitors as Anticancer Agents

In contrast to the three mTOR inhibitors discussed above, PI3K and Akt inhibitors are still at an early development phase, and so far no compound has reached the bedside. Despite this, three generations of compounds targeting PI3K have already been developed over time.

### PI3K and dual PI3K/mTOR inhibitors

The first-generation of PI3K inhibitors included compounds like Wortmannin and LY294002, which were able to bind all class I PI3Ks, thus being called “pan-inhibitors.” These compounds have been widely used in pre-clinical models to better characterize this complex pathway. However, due to very poor pharmacokinetic properties, they were never fully developed as anticancer drugs for clinical use (55).

More recently, compounds with better pharmacokinetic properties have been developed and are currently being evaluated within clinical trials in several malignancies (55), including genitourinary cancers (56) and others. These second-generation inhibitors are characterized by greater and isoform-specific selective activity (55).

The third generation of compounds comprises the so-called “dual PI3K/mTOR inhibitors.” These were developed after consideration that the CAT sites of PI3K and mTOR share a high degree of sequence homology. The potential advantage of these novel compounds (an advantage which still has to be confirmed *in vivo*) is that they inhibit not only all PI3K class I isoforms, but also mTORC1 and (more notably) mTORC2. In theory, this combined activity would lead to the strongest inhibition of the whole PI3K/Akt/mTOR pathway (57).

A list of PI3K inhibitors in pre-clinical and clinical development is reported in Table 2.

**Table 2:** **Phosphatidylinositol-3-kinase and “dual PI3K and mTOR” inhibitors in development [modified from ref. (53)]**.

Group	Selectivity	Compound/company/route of administration	Main feature(s)	Ongoing trials in
Pan-class I	Class I PI3K	GDC-0941 (Roche/Genentech) oral	Proved able to synergize with different agents (e.g., rapamycin, docetaxel, HER-targeting agents)	Breast, NHL, NSCLC
		BKM120 (Novartis) oral	Peculiar ability to penetrate the blood-brain barrier	Breast, colo-rectal, endometrial, GIST, leukemia, melanoma, NSCLC, pancreatic, RCC, transitional cell carcinoma, squamous cell carcinoma of the head and neck
		PX866 (Oncothyreon) oral	Proved able to synergize with chemotherapy, radiation, and targeted agents (e.g., EGFR inhibitors)	Colo-rectal, glioblastoma, NSCLC, squamous cell carcinoma of the head and neck
Isoform-specific	PI3Kα	GDC-0032 (Roche/Genentech) oral	Sparing the β-isoform of PI3K, it may reduce some undesired adverse events, e.g., metabolic abnormalities	Different solid cancers
	PI3Kβ	GSK2636771 (GSK) oral	Studied especially in patients whose tumors lack PTEN expression	Different solid cancers
	PI3K-γ and -δ	IPI-145 (Infinity) oral	Since PI3K-γ and -δ isoforms are preferentially expressed in leukocytes, where they have distinct and non-overlapping roles in key cellular functions (e.g., cell proliferation, differentiation, migration, and immunity) it may be particularly active in hematological malignancies (as well as in inflammatory diseases)	Different hematological malignancies
	PI3Kδ	CAL-101 (Gilead sciences) oral	Since PI3K-δ is preferentially expressed in leukocytes, may be particularly active in hematological malignancies; furthermore, the targeted inhibition of PI3K-δ is designed to preserve PI3K signaling in normal cells	AML, CLL, HL, NHL, multiple myeloma
Dual PI3K/mTOR	PI3K and mTOR	NVP-BEZ235 (Novartis) oral	This drug also potently inhibits ATM and DNA-PKcs, the two major kinases responding to ionizing radiation-induced DNA double-strand breaks, resulting in significant attenuation of double-strand breaks repair. May thus be developed as a radiosensitizer. Also the first PI3K inhibitor to enter clinical trials, in 2006; issues in its bioavailability are presently hampering its development	Breast, RCC

*NHL, non-Hodgkin’s lymphoma; NSCLC, non-small cell lung cancer; GIST, gastro-intestinal stromal tumor; RCC, renal cell carcinoma; AML, acute myeloid leukemia; CLL, chronic lymphocytic leukemia; HL, Hodgkin’s lymphoma*.

### Akt inhibitors

So far, compounds that target Akt ATP binding site, its PH domain, LINK, and the protein substrate sites have been developed (12, 58). Compared to PI3K, and especially mTOR inhibitors, fewer Akt-targeting agents have entered clinical development (58), even though one of them, Miltefosine, has already completed a phase III trial (59). A list of Akt inhibitors under pre-clinical and clinical development is given in Table 3.

**Table 3:** **Akt inhibitors in development**.

Compound/drug (company)	Characteristics	Clinical development
Miltefosine (Zentaris GmbH)	As 6% topical solution, proved able to increase time to treatment failure (in a double-blind, placebo-controlled, phase III trial) in cutaneous metastases from breast cancer patients Registered and used in India, Colombia, and Germany for the treatment of visceral and cutaneous leishmaniasis Targets HIV-infected macrophages. The HIV protein Tat activates pro-survival PI3K/Akt pathway in primary human macrophages. Miltefosine acts by inhibiting the PI3K/Akt pathway, thus removing the infected macrophages from circulation, without affecting healthy cells	
Perifosine (Keryx/Aeterna Zentaris)	Orally active alkyl-phosphocholine compound Modulates membrane permeability, membrane lipid composition, phospholipid metabolism, and mitogenic signal transduction, resulting in cell differentiation and inhibition of cell growth Inhibits the anti-apoptotic mitogen-activated protein kinase (MAPK) pathway and modulates the balance between the MAPK and pro-apoptotic stress-activated protein kinase (SAPK/JNK) pathways, thereby inducing apoptosis	Stopped after several phase II studies
MK2206 (Merck/Astra Zeneca)	Orally bioavailable allosteric inhibitor of the serine/threonine protein kinase Akt (protein kinase B) Binds to and inhibits the activity of Akt in a non-ATP competitive manner, which may result in the inhibition of the PI3K/Akt signaling pathway and tumor cell proliferation and the induction of tumor cell apoptosis	Phase I and II trials ongoing as single-agent or in combination with other drugs – e.g., chemotherapeutic, hormonal, and other targeted agents
RX-0201 (Rexahn pharmaceuticals)	A 20-mer antisense oligodeoxynucleotide directed against Akt Binds to Akt-1 mRNA, inhibiting translation of the transcript; suppression of Akt-1 expression may result in the inhibition of cellular proliferation, and the induction of apoptosis in tumor cells that overexpress Akt-1	Phase II study in combination with gemcitabine in pancreatic cancer closed
Erucylphosphocholine (a.k.a. ErPC or AEZS-127) Aeterna Zentaris	Structurally related to Perifosine, it inhibits Akt, but also impacts other signaling pathways (most prominently, Raf-MEK-ERK) Intravenous use	Currently under pre-clinical development
PBI-05204 (a.k.a. Oleandrin) (Phoenix biotechnology)	A lipid soluble cardiac glycoside derived from *Nerium oleander* Specifically binds to and inhibits the α3 subunit of the Na/K-ATPase pump in human cancer cells. This may inhibit the phosphorylation of Akt, upregulate MAPK, inhibit NF-κb activation, and inhibit FGF-2 export and may downregulate mTOR thereby inhibiting p70S6K and S6 protein expression, ultimately resulting in the induction of apoptosis As cancer cells with relatively higher expression of the α3 subunit and with limited expression of the α1 subunit are more sensitive to oleandrin, one may predict the tumor response to oleandrin based on the tumors Na/K-ATPase pump protein subunit expression	Early clinical development
GSK690693 (GSK)	An aminofurazan-derived inhibitor of Akt kinases 1, 2, and 3 May also inhibit other protein kinases including protein kinase C (PKC) and protein kinase A (PKA)	Early clinical development
XL-418 (Exelixis)	A dual inhibitor of Akt and p70S6K	Enhance apoptosis in combination with XL647, an inhibitor of multiple receptor tyrosine kinases including EGFR, HER2, and VEGFR, in pre-clinical tumor models In a phase I study, low drug exposure was achieved and the trial was thus stopped

## Mechanisms of Resistance to PI3K/Akt/mTOR Inhibitors

Despite all the successes (achieved with mTOR inhibitors) and expectations (related to novel anti-PI3K and Akt drugs), none of the above drugs is currently able to cure a single patient with cancer.

As with all antineoplastic agents, this is mainly due to the development of resistance. The underlying molecular basis of resistance, either intrinsic or acquired, remains largely unknown and has not been well characterized. So far, multiple mechanisms of resistance to targeted agents have been proposed, including secondary target mutations, activation of alternative, parallel, signaling pathways, and amplification of downstream alterations within the same pathway (60).

Resistance to mTOR inhibitors has been at least partially clarified. Indeed, it is often linked to different negative feedback loops. In one loop, mTORC1 inhibition leads to upregulation of receptor tyrosine kinases (RTKs or substrates) such as platelet-derived growth factor receptors (PDGFRs) and insulin receptor substrate-1 (IRS-1), resulting in increased PI3K-dependent Akt phosphorylation at Ser473. In another loop, mTORC1 inhibition leads to PI3K-Ras activation, which leads to an increase in MAPK signaling (61–63).

Furthermore, aberrant activation of MYC may contribute to acquired resistance to PI3K/Akt/mTOR-targeted therapy. Indeed, targeting this pathway may cause MYC activation through PDK1-dependent MYC phosphorylation and MYC amplification, which is parallel to PIK3CA-dependent Akt and MAPK activation, thus attenuating the therapeutic effect of PI3K/Akt/mTOR inhibitors (64–66).

Finally, a recent report analyzed SGK (serum- and glucocorticoid-regulated kinase) levels and the relative sensitivity of a panel of breast cancer cells toward two distinct Akt inhibitors (67). This study showed a number of Akt-inhibitor-resistant lines displaying markedly elevated SGK1 that also exhibited significant phosphorylation of the SGK1 substrate NDRG1 [neuroblastoma-derived Myc (N-Myc) downstream-regulated gene 1]. In contrast, most Akt-inhibitor-sensitive cell lines displayed low or undetectable levels of SGK1. Intriguingly, despite low SGK1 levels, several Akt-inhibitor-sensitive cell lines showed marked NDRG1 phosphorylation that, unlike resistant cells, were suppressed by Akt inhibitors. Furthermore, SGK1 knockdown markedly reduced proliferation of Akt-inhibitor-resistant cells, but not Akt-sensitive cells (67). Taken together, these results clearly suggest that SGK1 levels, as well as responses of NDRG1 phosphorylation to Akt inhibitor administration, could help us predict the sensitivity or resistance of tumor cells to Akt-targeting drugs.

Autophagy may represent another mechanism of resistance from Akt/mTOR targeting. Indeed, autophagy induction proved able to protect MCL cells from Akt/mTOR inhibition. Furthermore, selective triple knockdown of the autophagy genes ATG7, ATG5 and ATG3, and pre-treatment with the autophagy inhibitor hydroxychloroquine, efficiently overcame the resistance to Akt/mTOR inhibitors in this model, leading to the activation of the mitochondrial apoptotic pathway (68, 69). Taken together, these results suggest that counteracting autophagy may represent an attractive strategy for sensitizing lymphoma cells to everolimus-based therapy. Furthermore, autophagy facilitates cancer cell resistance also to cytotoxic chemotherapy and radiation treatment (70).

## Conclusion

The PI3K/Akt/mTOR pathway represents a good example of the concept of redundancy in biological systems, particularly in cancer cells. Indeed, cancer responds to chronic treatment with drugs targeting a single pathway by adapting its signaling circuitry, taking advantage of pathway redundancy and routes of feedback and crosstalk to maintain their function and thus escape from drug-induced growth inhibition and death (71–73).

That is why, despite recent successes (achieved in completely different diseases such as kidney and breast cancer, pNETs, and other malignancies), tumors ultimately evade inhibition of this pathway.

Novel agents targeting PI3K/Akt/mTOR promise further improvement of the results achieved so far through higher selectivity and potency, as well as to combinability with other therapeutic strategies. However, only translational research, addressing this variegated and complex network of highly integrated signaling pathways and mechanisms of resistance to their inhibition, will be able to help us take another step forward.

## Author Contribution

All the authors equally contributed to the preparation of this manuscript.

## Conflict of Interest Statement

The authors declare that the research was conducted in the absence of any commercial or financial relationships that could be construed as a potential conflict of interest.
